# Can the Ability to Play Steady Beats Be Indicative of Cognitive Aging? Using a Beat Processing Device

**DOI:** 10.3390/bs14111113

**Published:** 2024-11-20

**Authors:** Hyun Ju Chong, Jin Hee Choi, Ga Eul Yoo

**Affiliations:** 1 Department of Music Therapy, Graduate School, Ewha Womans University, Seoul 03760, Republic of Korea; hju@ewha.ac.kr; 2 Ewha Music Wellness Research Center, Ewha Womans University, Seoul 03760, Republic of Korea; mtgenie@ewha.ac.kr

**Keywords:** rhythm idiom, temporal processing, rhythm reproduction, cognitive aging, older adults

## Abstract

This study aimed to examine whether different rhythm idioms significantly affect the reproduction accuracy of older adults and whether the participants’ age and personal current engagement in music affect their ability to reproduce rhythm. A total of 79 older adults participated in the study. Participants were required to reproduce six different rhythm idioms, and their accuracy in rhythm reproduction was measured using the R index. The data were analyzed considering the participants’ age sub-group and current engagement in music. The findings showed differences in reproduction accuracy across various rhythm idioms, particularly in relation to steady recurring notes and dotted notes with different intervals. The highest reproduction accuracy was found for the isochronous beat pattern, while the rhythm idiom starting with longer intervals yielded the lowest accuracy. Age and current personal engagement in music did not significantly affect rhythm performance. However, the study identified a significant correlation between decreased accuracy in reproducing a steady rhythm and diminished general cognitive ability. This study indicates that rhythm performance can be indicative of cognitive abilities related to temporal information processing. The findings support the potential use of rhythm tasks to evaluate cognitive performance in older adults with varying cognitive levels.

## 1. Introduction

There has been a growing emphasis on promoting healthy aging within modern society. A prominent area of research in music therapy is the use of music to maintain and potentially enhance cognitive function in older adults [1,2]. Particularly, music interventions that involve active engagement, such as playing musical instruments, incorporate a range of multi-dimensional skills, including working memory, executive control of attention, motor functions, and sensory-motor synchronization [3,4]. By engaging these integrative skills, music interventions can effectively optimize the residual abilities of older adults during the critical period of rehabilitative treatment [5,6].

Among the tasks related to instrument playing, rhythm playing stands out as it actively involves both cognitive and motor functions. Previous research has shown that rhythm performance, such as rhythm production or reproduction, relies on the ability to retain and process temporal sequences based on internal timing structures, which require attention and memory [7,8]. The cognitive demands of rhythm-playing tasks vary depending on the complexity of the rhythm patterns, the combination of patterns, and the variability in intervals between beats, ranging from simple to complex [9,10].

As aging progresses, temporal processing abilities tend to decline, and this decrease is also observed in rhythm reproduction ability. The internal timing of older adults becomes slower than that of younger adults when engaging in spontaneous hand tapping without external cues [11,12]. Their speed of movement also decreases when attempting to tap as rapidly as possible [13]. Changes in temporal information processing may also impact the maintenance of internal beats [14]. Furthermore, greater task complexity, such as recreating rhythm patterns with unequal beats and requiring higher-level executive functioning, is associated with increased variability in rhythm reproduction performance [15]. Considering the well-documented association between decreased temporal processing and aging [8], rhythm reproduction holds promise as an alternative means of assessment [7,16].

Previous research has indicated that the level of rhythm performance can reflect varying degrees of decline or preserved temporal processing and perceptual-motor abilities in older adults [17]. This effort focuses on the construction and combination of rhythm patterns using different intervals between beats, including shorter versus longer intervals, and small integer ratios versus larger or non-integer ratios [4,18]. The findings indicate the involvement of distinct cognitive mechanisms, engaging different levels of attentional resources, explicit efforts, and memory in processing rhythm patterns with varying intervals [19,20]. The evidence suggests that rhythm patterns with shorter durations or simpler ratios (e.g., 1:2 or 1:3 ratios) involve fewer cognitive demands, allowing older adults to more easily utilize their preserved temporal abilities without heavily relying on higher-order cognitive functions. In contrast, rhythm patterns with longer durations and more complex ratios require increased attentional resources, thereby highlighting age-related declines.

Given the potential benefits of a systematic approach to constructing rhythm patterns for assessing older adults, this study selected basic rhythm idioms aimed at introducing varying levels of difficulty while maintaining the same rhythmic structure but systematically altering the arrangement or sequence of the idioms. By using rhythm idioms and maintaining the musicality of the constructed rhythm patterns while avoiding random arrangements of ratios, we aimed to improve their applicability and relevance for real intervention settings. Moreover, our investigation aimed to explore the impact of individual factors on rhythm performance through a comparative analysis involving two distinct age sub-groups: the young-old and older-old groups. By employing this specific categorization of age groups, we aimed to address potential confounding effects resulting from treating a wide age range as a homogeneous population. This approach enabled us to determine whether a specific stage of aging might lead to distinct effects on temporal processing within the older population. Additionally, we compared participants with current music engagement to those without such experiences. Including music engagement as a factor helped control potential biases related to differences in musical literacy and familiarity with the assigned rhythm task.

Therefore, the purpose of this study was to examine whether there are differences in rhythm reproduction accuracy among older adults. This study further investigated whether age subgroups and current music engagement influenced their rhythm reproduction abilities. The research questions addressed in this study were as follows:

1. Does the type of rhythm idioms significantly affect the reproduction accuracy of older adults?
2. Are the participants’ age or current involvement in musical activities associated with their rhythm reproduction accuracy?
3. Can rhythm performance accuracy, along with age, explain the general cognitive level as indicated by MMSE-K?

## 2. Materials and Methods

### 2.1. Participants

This study’s procedures and ethical issues were approved by the Institutional Review Board (IRB) of Ewha Womans University (IRB No. 162-16). Participants were recruited from elderly care facilities. The following inclusion criteria were applied: individuals aged between 60 and 90 years, with no hearing difficulties or history of neurological impairment. Moreover, participants were required to achieve a minimum score of 24 on the Korean version of the Mini-Mental State Examination (MMSE-K) [21], indicating the absence of cognitive impairment. Written informed consent forms were obtained from each individual prior to their participation in the study. The experimental sessions took place individually in a quiet and private setting within the facilities. Out of the 85 older adults who initially responded to the flyers posted, 79 met the inclusion criteria and were included in the study. For the sub-analysis of participants based on age and current music engagement, demographic information, including gender, age, and current music engagement, was collected upon their agreement to participate in the study. To evaluate current music engagement, participants were asked whether they were involved in activities focused on specific musical experiences, such as choir participation, singing classes, musical instrument lessons, or other forms of active music involvement, either individually or through community programs for older adults. The types of activities were not specified, as many included a combination of various musical behaviors, such as singing combined with listening to music and playing instruments while singing. Detailed demographic information of the participants is presented in Table 1.

### 2.2. Rhythm Idioms for the Rhythm Reproduction Task

In this study, the rhythm reproduction task involved the use of different types of rhythm idioms, each lasting for the duration of four beats. Previous studies indicate that the complexity of rhythm reproduction tasks is determined by interval length and ratio [19,20]. To investigate temporal processing in older adults with varying levels of musical experiences, this study considered stimuli constructed using variations in simple ratios (1:2 or 1:3) within regular beat patterns were considered for stimuli construction. The use of these idioms for older adults, including those with potential cognitive impairments, were validated by a prior study [9]. These idioms included isochronous beats (pulsation), subdivided notes, as well as combinations of longer and shorter notes (e.g., dotted or extended notes and eighth note with the ratio of 3:1 or 1:3). Each rhythm idiom was constructed to repeat a two-beats of a smaller rhythm unit. The basic beat interval used for all rhythm idioms was set at 1000 milliseconds as demonstrated in Rhy 1 in Table 2. Specifically, the basic beat in Rhy1 consisted of interstimulus intervals (ISI) of 1000 milliseconds, while subdivided notes consisted of ISIs of 500 milliseconds. The details of the six rhythm idioms are displayed in Table 2.

### 2.3. Measurement Tool: Beat Processing Device

**Figure 1:**
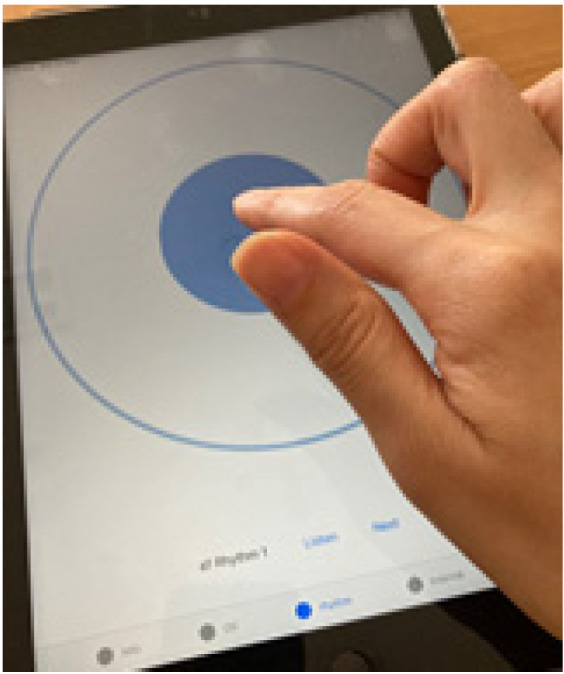
Illustration of using BPD for rhythm reproduction tasks.

To collect and record participants’ rhythm tapping data, an investigator-developed iPad application called the Beat Processing Device (BPD) was used (Figure 1). The BPD is a mobile application designed specifically for measuring and quantifying the accuracy of rhythm reproduction. It serves two primary functions: providing rhythm tasks in a consistent and objective manner and accurately measuring and calculating data relevant to rhythm reproduction, including tapping onset and duration. Previous research [9] has validated the applicability of this device for use with older adults.

The BPD randomly presented the six rhythm idioms for each participant. Following a practice trial, participants were instructed to tap on the iPad screen using their dominant hand. To assess participants’ rhythm reproduction ability (indicated by R), the accuracy of performance was analyzed using the following formula [22].

$$R=\sum_{i=1}^{n}\frac{\frac{I_{si}}{T_{S}}-\frac{I_{i}}{T}}{\frac{I_{i}}{T}}\cdot \frac{1}{n}$$

The formula for measuring the accuracy of rhythm reproduction task performance is as follows. The value of R represents the ratio of the intervals in the rhythm idioms reproduced by the participant. In the formula, ‘*i*’ represents the index of the interval between two consecutive events. ‘*I_i_*’ denotes the temporal duration of the *i*-th interval in the standard rhythmic pattern, ‘*I_si_*’ represents the corresponding performed temporal interval, and ‘*T*’ signifies the total duration of each rhythm idiom. A lower value of R indicates a higher level of accuracy in reproducing the rhythm.

### 2.4. Data Analysis

To examine the rhythm performance across different rhythm idioms and subgroups, specifically, age subgroups consisting of the young-old group (participants aged 60–74) and the older-old group (participants aged 75–90), as well as current involvement in musical activity (music involvement versus non-involvement), two sets of mixed models of repeated measures analysis of variance (ANOVA) were conducted. For the first analysis, age subgroups were used as the between-subject factor, and the R index of rhythm reproduction accuracy as the within-subject factor. In the second analysis, current involvement in musical activity was the between-subject factor, with the rhythm idiom remaining as the within-subject factor.

Additionally, this study aimed to investigate the correlation between rhythm performance and cognitive states, while considering the effect of age. To achieve this, the relationship between rhythm performance, as indicated by the R index, and general cognitive level, as indicated by the MMSE-K, was assessed using Pearson’s correlation. For the rhythm idiom that was found to be significantly correlated with the MMSE-K, a multiple regression analysis was conducted using the R index and age as factors. All statistical analyses were performed using SPSS 27.0 (IBM, Armonk, NY, USA).

## 3. Results

### 3.1. Rhythm Reproduction Accuracy Across Different Rhythm Idioms

The study investigated the differences in rhythm reproduction ability across various rhythm idioms. Analysis of the R index values for each rhythm idiom (Table 3) showed that the idiom consisting of pulsation (Rhy1) exhibited the highest accuracy, followed by Rhy3 and Rhy2, which incorporated subdivided notes. Rhy5, which began with a shorter note, demonstrated the second highest accuracy. The two idioms starting with longer notes, Rhy4 and Rhy6, displayed the lowest accuracy.

The results of a repeated measures ANOVA indicated a significant difference in the rhythm reproduction accuracy index, as measured by the R value, among the rhythm idioms, *F*(5, 390) = 25.542, *p* < 0.001. Post hoc tests with Bonferroni’s correction showed that the R index of Rhy1 was significantly lower than that of all other rhythm idioms (*p* < 0.05), indicating the highest accuracy. However, Rhy3 had a similar level of accuracy and did not significantly differ from Rhy1. Additionally, the R index for idioms ranked second to fifth in terms of accuracy was significantly lower than those ranked lower, except for the idiom directly below them (e.g., Rhy3 had a significantly lower R index than Rhy5, Rhy4, and Rhy6, but not Rhy2, see Table 4). There was no significant difference in the R index between Rhy4 and Rhy6, both of which had the highest R indices. Of particular interest is the comparison between rhythm idioms with an identical basic rhythm unit but differing in the order, as seen in Rhy3 versus Rhy4 and Rhy5 versus Rhy6. These paired idioms varied solely based on whether either shorter notes or longer notes preceded them. Notably, the rhythm idiom featuring shorter notes followed by longer notes exhibited significantly higher accuracy (e.g., Rhy3 > Rhy 4, Rhy5 > Rhy6).

### 3.2. Rhythm Reproduction Accuracy Depending on the Age Sub-Group and Current Engagement in Music

The descriptive statistics of the R indices and the differences in these indices depending on the participants’ age sub-group and current engagement in music are displayed in Table 3. When the age sub-group was set as a between-subject factor and the rhythm idiom served as a within-subject factor, the results of a mixed model of repeated measures ANOVA showed a significant rhythm idiom effect, *F*(5, 385) = 24.297, *p* < 0.001, indicating that different rhythm idioms had varying effects on rhythm reproduction accuracy. However, there was no significant effect of age group, *F*(5, 385) = 0.863, *p* = 0.356, and the interaction effect between age group and rhythm idiom was also not significant, *F*(5, 385) = 0.784, *p* = 0.562. These findings indicate that age sub-groups exhibited similar trends in performing the rhythm reproduction task across different rhythm idioms.

**Figure 2:**
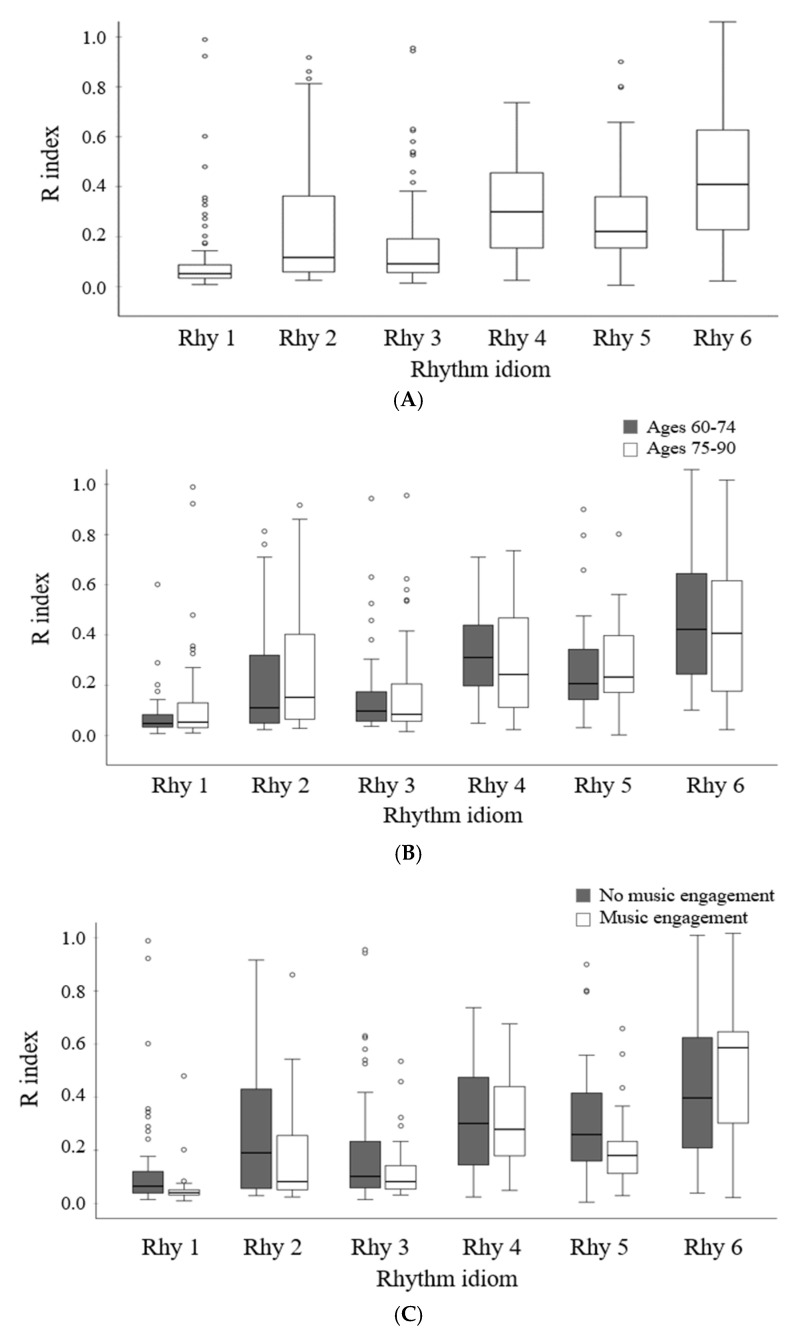
The R index for each rhythm idiom. The top panel (**A**) indicates the R index for each rhythm idiom. The panels (**B**,**C**) illustrate the indices depending on the age group and current engagement in music, respectively.

Regarding the rhythm idiom factor, the post hoc analysis (see Table 4) showed statistically significant differences in all comparison pairs, except for five pairs that were consistent with the total group comparison. Notably, these five pairs did not indicate significant differences with the directly lower idiom (i.e., Rhy1-Rhy3, Rhy3-Rhy2, Rhy2-Rhy5, and Rhy4-Rhy5). These rhythm idioms (Rhy2, Rhy5, and Rhy4) ranked third to fifth, respectively, in terms of the R index order (Figure 2). Therefore, the lack of significant differences between these idioms indicates no differences among idioms with a middle level of rhythm reproduction accuracy. However, the R index for Rhy6 which had the highest value (indicating the lowest accuracy), was significantly different from all other idioms (*p* < 0.05).

Regarding the effect of current engagement in music, it did not yield a significant effect, *F*(5, 385) = 3.533, *p* = 0.064, whereas the effect of rhythm idioms was significant, *F*(5, 385) = 26.847, *p* < 0.001. The interaction effect between current engagement in music and rhythm idiom was not significant, *F*(5, 385) = 1.796, *p* < 0.113. The post hoc analysis for the rhythm idiom factor (see Table 4) indicated findings similar to the age subgroup analysis, where all comparison pairs, except for five pairs (i.e., Rhy1-Rhy3, Rhy3-Rhy2, Rhy2-Rhy5, Rhy2-Rhy4, and Rhy4-Rhy5), showed statistical significance (see Table 4 and Figure 2).

### 3.3. Rhythm Performance as a Predictor of Cognitive Ability

Lastly, a Pearson’s correlation was conducted to examine the relationship between the R index of each rhythm idiom and cognitive ability measured by MMSE-K. The results are presented in Table 5. The results showed a significant correlation between the R index of Rhy1, a regular-paced beat pattern, and MMSE-K (*r* = −0.258). This negative coefficient indicates that as the R index of Rhy1 decreased, indicating increased accuracy in rhythm reproduction, the MMSE-K score increased. To further investigate the relationship between the R index of Rhy1 and the MMSE-K score, a simple linear regression analysis was conducted. The analysis demonstrated nonsignificant multicollinearity with a VIF of 1.0, and the model accounted for 12.2% of the variance (*R*^2^ = 0.122). The overall regression was statistically significant, *F*(1, 77) = 5.269, *p* = 0.007. The R index of Rhy1 was found to be a significant predictor of general cognitive ability, as measured by the MMSE-K, *β* = −2.225, *p* = 0.049. Age was also a significant predictor, *β* = −0.066, *p* = 0.032. The analysis indicated that an increase in one unit in the R index of Rhy1, representing reduced rhythm reproduction accuracy, significantly predicted a 2.2-point decrease, in general, cognitive ability as measured by the MMSE-K. Additionally, an increase in age by one year predicted a 0.6-point decrease in MMSE-K score.

## 4. Discussion

This study investigated the performance of older adults in reproducing rhythm idioms across varying rhythm idioms and examined the influence of age and current engagement in musical activities on this performance. The study focused on examining temporal processing in relation to rhythm reproduction, an area of particular relevance in understanding cognitive processes in older adults. The results supported that older adults exhibit varying levels of accuracy in reproducing rhythm depending on the rhythm idiom used. The rhythm idiom consisting of an isochronous sequence with repetitive pulsation (i.e., Rhy1) demonstrated the highest accuracy, compared to idioms involving subdivisions and combinations of short and long intervals. This finding aligns with previous research, indicating that beat-based patterns are automatically processed and require less cognitive demand [23]. Therefore, the ability to reproduce repetitive and regularly paced rhythm tends to be relatively preserved in older adults compared to more complex rhythm patterns [13].

Research in the context of rhythm idioms with varying cognitive demands for temporal processing supports that the length of the interval influences cognitive involvement. Shorter intervals engage more automatic processing without explicit cognitive involvement, while longer intervals require higher cognitive processing [24]. The findings from the current study are consistent with previous findings. Rhy1 (pulsation) demonstrated significantly higher accuracy than Rhy3 (subdivided beat pattern). The rhythm idioms with a ratio of 1:3 (Rhy5) or 1:1:2 (Rhy3) exhibited significantly higher accuracy compared to patterns with a ratio of 3:1 (Rhy6) or 2:1:1 (Rhy4). Consistent with these results, Rhy6 showed the lowest accuracy, supporting the notion that longer intervals demand increased attentional resources. Reproducing such rhythm idioms involves the ability to maintain temporal information over a longer duration, which engages attention and working memory processes [25]. Additionally, reproducing rhythm with longer intervals or different combinations of time intervals may require inhibitory control, which is closely associated with complex and flexible attentional processing. Previous studies have also shown that motor reproduction errors increase when rhythm idioms with longer ISI are reproduced [25]. These findings highlight the importance of preserving temporal distances between beats (intervals) and the presence of a fixed temporal structure for enhancing accuracy in rhythm reproduction. These findings have practical implications for designing rhythm tasks that facilitate cognitive processing in older adults and for adjusting the complexity of rhythm sequences for different purposes.

Secondly, the study examined age sub-groups and current musical engagement as potential factors influencing task performance in the older population. In terms of age sub-groups, including the young-old age group (60–74) and the older-old age group (75–90), there was no significant effect of age group, while a significant difference among rhythm idioms was observed. These findings indicate that the aging process itself does not significantly contribute to differences in rhythm reproduction ability within this population. Previous studies comparing young adults with older adults in terms of rhythm ability have demonstrated a decline in rhythm task performance with aging, particularly concerning irregular rhythm patterns with varying interval ratios [6,26]. However, beyond the age of 50, differences in rhythm task performance appear to become less pronounced among age sub-groups [26], highlighting the greater influence of individual differences in cognitive processing on determining rhythm performance as individuals advance in age.

Furthermore, this study did not identify a significant effect of current music engagement on rhythm performance. Given that the older population exhibits greater heterogeneity in terms of music education or musical background across the population, it was necessary to control for varying levels of familiarity with the musical context or musical task when analyzing rhythm performance in older adults participating in this study. Meanwhile, previous research has suggested that active and intensive participation in music activities can enhance cognitive processing in the older population [27], but this finding was not supported by the current study. The lack of a significant effect of musical engagement in the current study may be attributed to the fact that the study solely analyzed whether participants were currently engaging in music activities, without systematically examining the type, duration, or frequency of music engagement. Future research should comprehensively investigate the relationship between music engagement and rhythm performance by considering the impact of different types, durations, and frequencies of musical engagement on rhythm tasks. Additionally, accounting for the varying cognitive demands associated with different musical activities would facilitate a more thorough comprehension of how musical engagement influences rhythm abilities in older adults.

Lastly, in an effort to determine whether a specific rhythm idiom could be indicative of the cognitive state of older adults, this study found a significant correlation between the accuracy of reproducing the steady beat pattern of Rhy1 and general cognitive ability, as measured by the MMSE-K. Furthermore, accurate reproduction of this beat-patterned rhythm (Rhy 1) was found to predict general cognitive function, with a 2.2-point decrease in the MMSE-K score predicted for each unit increase in accuracy indicated by a lower R index. These findings are consistent with previous research indicating that cognitive decline is associated with reduced attentional and motor control linked to internal timing and synchronization to external temporal cues [9,19]. The results indicate that the ability to accurately process and reproduce steady beat patterns reflects timing performance involving speed and accuracy of information processing [28,29]. Furthermore, given that MMSE-K measures general cognitive levels with simple assessment methods suitable for easily screening the older population, while not providing detailed assessments of specific cognitive abilities, isochronous tapping could be considered potential for indicating certain aspects of cognitive function in the older population. However, it is important to note that this study solely focused on healthy older adults, and further research is necessary to examine how rhythm reproduction ability may vary in individuals with varying levels of cognitive ability or impairment. Such research could provide valuable insights into diverse approaches for assessing cognitive aging within a musical context. Furthermore, future research could investigate the predictive role of active engagement in music for maintaining or enhancing cognitive ability in individuals with cognitive decline.

## 5. Conclusions

In conclusion, this study demonstrates that the accuracy of rhythm reproduction in older adults is influenced by the complexity and arrangement of rhythm idioms, with isochronous sequences incorporating repetitive pulsation being the most accurately reproduced. Additionally, rhythm idioms with shorter notes preceding longer notes showed higher accuracy for rhythm production with varying intervals. Regarding age sub-groups and current engagement in music, no significant effects were observed on rhythm performance, suggesting that the participating older adults in this study demonstrated a homogeneous condition in performing rhythm tasks overall. More importantly, a significant correlation was found between decreased accuracy in reproducing steady rhythm and reduced general cognitive ability. This suggests that a steady beat pattern can be a simple yet crucial indicator for temporal processing in older adults, supporting the notion that rhythm performance holds promise as a tool for assessing cognitive ability, particularly in tasks involving the speed and accuracy of information processing. To gain a comprehensive understanding of the relationship between rhythm performance and cognitive ability in older adults, future research should explore the impact of different types of musical engagement on rhythm tasks and examine varying levels of cognitive decline. Such investigation would also provide valuable implications for the potential benefits of music-based assessment and intervention in the context of cognitive aging.

## Figures and Tables

**Table 1:** Participants’ demographic information.

Variable	Total Group (*N* = 79)	Age Sub-Group		Current Music Engagement	
		Ages 60 to 74 (*n* = 44)	Ages 75 to 90 (*n* = 35)	Yes (*n* = 29)	No (*n* = 50)
Gender, Female, *n* (%)	59 (74.7%)	36 (81.8%)	23 (63.9%)	25 (86.2%)	34 (68.0%)
Age in years, M (SD)	73.8 (6.4)	69.1 (3.9)	79.8 (3.0)	70.8 (6.9)	75.6 (5.4)
MMSE-K, M (SD)	27.2 (1.8)	27.7 (1.7)	26.7 (1.7)	27.6 (1.8)	27.0 (1.7)

Note: F: female; M: male; MMSE-K: Korean version of Mini-Mental State Examination.

**Table 2:** Rhythm idioms used for the rhythm reproduction task.

Rhythm Idiom		Inter-Onset-Intervals (ms)	Ratio of Each Beat Interval	Components of Musical Notation
Rhy1	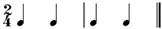	1000, 1000	1:1	Basic steady notes (pulsating beats)
Rhy2	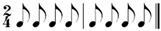	500, 500, 500, 500	1:1:1:1	Subdivided notes (eighth notes)
Rhy3	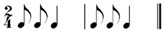	500, 500, 1000	1:1:2	Subdivided notes followed by longer note (basic interval)
Rhy4	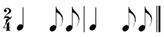	1000, 500, 500	2:1:1	Basic note followed by subdivided notes
Rhy5	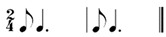	500, 1500	1:3	Subdivided shorter note (eighth note) followed by longer duration (extended beat)
Rhy6	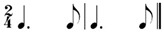	1500, 500	3:1	Dotted quarter note (extended beat) followed by a single short note (eighth note)

**Table 3:** The R index for each rhythm idiom depending on age sub-group and current engagement in music.

Rhythm Idiom		Total Group (*N* = 79)	Age Sub-Group		Current Engagement in Music	
			Young-Old (Ages 60–74; *n* = 44)	Older-Old (Ages 75–90; *n* = 35)	Yes (*n* = 29)	No (*n* = 50)
		*M* (*SD*)	*M* (*SD*)	*M* (*SD*)	*M* (*SD*)	*M* (*SD*)
Rhy1	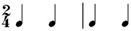	0.11 (0.17)	0.08 (0.10)	0.15 (0.23)	0.06 (0.09)	0.14 (0.20)
Rhy3	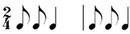	0.17 (0.20)	0.16 (0.18)	0.19 (0.22)	0.13 (0.12)	0.20 (0.23)
Rhy2	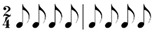	0.25 (0.26)	0.23 (0.26)	0.28 (0.27)	0.17 (0.19)	0.30 (0.29)
Rhy5	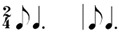	0.28 (0.23)	0.26 (0.19)	0.32 (0.26)	0.24 (0.22)	0.31 (0.23)
Rhy4	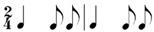	0.34 (0.24)	0.34 (0.21)	0.33 (0.26)	0.31 (0.18)	0.35 (0.26)
Rhy6	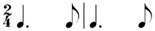	0.47 (0.29)	0.49 (0.28)	0.45 (0.30)	0.52 (0.32)	0.44 (0.28)

**Table 4:** Paired comparison of rhythm idioms for reproduction accuracy.

Group 1	Group 2	Total Group		Age Sub-Group Analysis		Current Music Engagement Subgroup Analysis	
		MD	*p*	MD	*p*	MD	*P*
Rhy1	Rhy2	−0.142	0.001 **	–0.140	0.002 **	–0.136	0.004 **
	Rhy3	–0.063	0.371	–0.060	0.489	–0.064	0.422
	Rhy4	–0.225	<0.001 ***	–0.220	<0.001 ***	–0.230	<0.001 ***
	Rhy5	–0.173	<0.001 ***	–0.172	<0.001 ***	–0.174	<0.001 ***
	Rhy6	–0.361	<0.001 ***	–0.355	<0.001 ***	–0.383	<0.001 ***
Rhy2	Rhy3	0.079	0.452	0.080	0.448	0.071	0.860
	Rhy4	–0.083	0.585	–0.080	0.727	–0.095	0.362
	Rhy5	–0.031	1.000	–0.032	1.000	–0.038	1.000
	Rhy6	–0.220	<0.001 ***	–0.215	<0.001 ***	–0.247	<0.001 ***
Rhy3	Rhy4	–0.162	<0.001 ***	–0.160	<0.001 ***	–0.166	<0.001 ***
	Rhy5	–0.110	<0.001 ***	–0.112	<0.001 ***	–0.109	0.001 **
	Rhy6	–0.298	<0.001 ***	–0.294	<0.001 ***	–0.318	<0.001 ***
Rhy4	Rhy5	0.052	1.000	0.048	1.000	0.057	1.000
	Rhy6	–0.137	0.007 **	–0.134	0.010 *	–0.152	0.003 **
Rhy5	Rhy6	–0.189	<0.001 ***	–0.183	0.001 **	–0.209	<0.001 ***

* *p* < 0.05. ** *p* < 0.01. *** *p* < 0.001.

**Table 5:** Correlation between R index and MMSE-K scores.

	R Index, *r* (*p*)					
	Rhy1	Rhy2	Rhy3	Rhy4	Rhy5	Rhy6
MMSE-K score	−0.258	−0.017	−0.011	0.013	0.000	−0.162
	(0.022 *)	(0.879)	(0.920)	(0.907)	(0.999)	(0.154)

* *p* < 0.05.

## Data Availability

The data are available on request from the corresponding author.
